# Predictors of Rehospitalization and Mortality in Diabetes-Related Hospital Admissions

**DOI:** 10.3390/jcm10245814

**Published:** 2021-12-12

**Authors:** Milena Kozioł, Iwona Towpik, Michał Żurek, Jagoda Niemczynowicz, Małgorzata Wasążnik, Yaroslav Sanchak, Waldemar Wierzba, Edward Franek, Magdalena Walicka

**Affiliations:** 1 Department of Analyses and Strategies, Polish Ministry of Health, 00-952 Warsaw, Poland; m.koziol@mz.gov.pl (M.K.); m.zurek@mz.gov.pl (M.Ż.); jagodaszmidt96@gmail.com (J.N.); m.wasaznik@mz.gov.pl (M.W.); 2 Department of Internal Diseases, Collegium Medicum, University of Zielona Góra, 65-046 Zielona Góra, Poland; itowpik@cm.uz.zgora.pl; 3 Doctoral School, Medical University of Warsaw, 02-091 Warsaw, Poland; 4 Department of Internal Diseases, Endocrinology and Diabetology Central, Clinical Hospital of the Ministry of the Interior and Administration in Warsaw, 02-507 Warsaw, Poland; sanchaky@gmail.com (Y.S.); edward.franek@cskmswia.gov.pl (E.F.); 5 Satellite Campus in Warsaw, University of Humanities and Economics in Lodz, 01-513 Warsaw, Poland; waldemar.wierzba@cskmswia.gov.pl; 6 Department of Human Epigenetics, Mossakowski Medical Research Institute, 02-106 Warsaw, Poland

**Keywords:** diabetes, readmission, rehospitalization, death, predictors

## Abstract

The risk factors of rehospitalization and death post-discharge in diabetes-related hospital admissions are not fully understood. To determine them, a population-based retrospective epidemiological survey was performed on diabetes-related admissions from the Polish national database. Logistic regression models were used, in which the dependent variables were rehospitalization due to diabetes complications and death within 90 days after the index hospitalization. In 2017, there were 74,248 hospitalizations related to diabetes. A total of 11.3% ended with readmission. Risk factors for rehospitalization were as follows: age < 35 years; male sex; prior hospitalization due to acute diabetic complications; weight loss; peripheral artery disease; iron deficiency anemia; kidney failure; alcohol abuse; heart failure; urgent, emergency, or weekend admission; length of hospitalization; and hospitalization in a teaching hospital with an endocrinology/diabetology unit. Furthermore, 7.3% of hospitalizations resulted in death within 90 days following discharge. Risk factors for death were as follows: age; neoplastic disease with/without metastases; weight loss; coagulopathy; alcohol abuse; acute diabetes complications; heart failure; kidney failure; iron deficiency anemia; peripheral artery disease; fluid, electrolytes, and acid–base balance disturbances; urgent or emergency and weekend admission; and length of hospitalization. We concluded that of all investigated factors, only hospitalization within an experienced specialist center may reduce the frequency of the assessed outcomes.

## 1. Introduction

Diabetes mellitus is a metabolic disease characterized by hyperglycemia. This condition is one of the fastest growing public health problems. According to the International Diabetes Federation (IDF) data, in 2021, the number of people with diabetes in the world has reached 537 million. This number is expected to increase to 643 million by 2030 and 783 million by 2045 [1].

Chronic hyperglycemia, in patients with poorly controlled diabetes, causes damage to various organs and systems and induces chronic diabetes complications that affect the patient’s quality of life. Such patients have higher readmission rates than patients without known diabetes [2]. Additionally, these readmissions are longer, and patients have higher in-hospital mortality and overall mortality than those without diabetes [3,4,5]. Diabetes-related rehospitalizations significantly increase the direct and indirect costs of healthcare [6].

Understanding predictors of rehospitalization and mortality would allow for a more focused care, potentially prevent readmission, and decrease the risk of premature death of high-risk patients. Limited data from literature indicate that comorbidities strongly influence the probability of a patient’s death [7,8]. Elixhauser et al. showed that in inpatients, comorbidities had independent effects on and were associated with longer hospitalization and mortality [9]. In turn, our previous study determined that, in patients who were hospitalized in nonsurgical departments, higher mortality was related to some factors associated with the hospitalization such as hospital type, type and day of admission, and length of hospital stay (comorbidities were not assessed) [10]. Likewise, the last published review [6] indicated that in the diabetes population, the problem of rehospitalization is complex because the risk factors of readmission can be related to the patient’s medical condition such as certain comorbidities, abnormal laboratory values, inpatient hypoglycemia, hyperglycemia and glucose variability, low exercise capacity, as well as factors not related to the patient’s medical condition such as insurance type, insulin treatment, length of hospitalization, and history of admissions. 

Due to the limited data, we explored the predictors of rehospitalization and death within 90 days of discharge in diabetes-related hospital admissions.

## 2. Materials and Methods

### 2.1. Study Population

The study was a population-based retrospective epidemiological survey. The analyses were performed using two major databases, the Digital Affairs—Chancellery of the Prime Minister and the National Health Fund (NHF). The database of the Digital Affairs—Chancellery of the Prime Minister contains information regarding the dates of death within the population. The NHF database consists of all reported hospitalizations, visits to an outpatient specialist, primary and psychiatric care, addiction treatment, as well as filled and reimbursed prescriptions. 

The only public healthcare payer in Poland is the National Health Fund, which records all medical procedures financed from public sources and maintains the national database of hospitalizations. NHF’s database contains medical and sociodemographic data, which include the diagnoses coded according to the International Classification of Diseases, 10th revision (ICD-10); the medical procedures coded using the International Classification of Diseases, 9th Revision (ICD-9); and patient personal information such as identification number (PESEL), age, sex, and place of residence. Lastly, the database also distinguishes the main diagnosis (the primary cause of hospitalization) from other accompanying diagnoses.

The data from the national database of hospitalizations of all patients who underwent hospitalization primarily related to diabetes in Poland between January 2017 and December 2017 were assessed. These diabetes-related hospitalizations were identified with the following ICD-10 codes: E10—Type 1 diabetes mellitus; E11—Type 2 diabetes mellitus; E12—Malnutrition-related diabetes mellitus; E13—other specified diabetes mellitus; E14—Unspecified diabetes mellitus.

### 2.2. Endpoints and Variables

For all patients who underwent a hospitalization due to diabetes (defined as the index hospitalization), two endpoints were analyzed. The first endpoint was readmission to the hospital at any point during a period of 90 days following discharge from the index hospitalization, and the second endpoint was death at any point during a period of 90 days following discharge from the index hospitalization. 

In order to assess the risk factors of rehospitalization, a logistic regression model was developed that compares rehospitalized patients and those not requiring rehospitalization. 

In order to assess the risk factors of death, a logistic regression model was developed that compares patients who died during the period of 90 days after discharge with those who survived this period. 

Based on the literature review, a number of potential risk factors were defined and partitioned into three main groups. These groups include risk factors in the patient demographic profile, patient medical history, and factors related to the index hospitalization. A description of the variables used in this study is presented in Table 1, Table 2 and Table 3.

### 2.3. Inclusion and Exclusion Criteria

The inclusion criterium for this study was hospitalization in 2017 that was primarily related to diabetes. For the analysis of the risk of rehospitalization within 90 days after discharge, the exclusion criteria were death of a patient up to 90 days after discharge and death during the index hospitalization. For the analysis of risk of death within 90 days after discharge, the exclusion criterium was death during the index hospitalization.

### 2.4. Statistical Analysis

The statistical analysis included logistic regression models in which the dependent variables were rehospitalization and death at any point during a period of 90 days after the index hospitalization. The analyses of variance inflation factors (VIF) and Pearson and Spearman rank correlation coefficients were performed to detect collinearity of any monotonic dependencies (including nonlinear). Kaplan–Meier curves were used to visualize the results of both analyses.

In order to identify the model that is best suited for these data, and to extract the explanatory variables having the greatest impact on the explained variable, the stepwise selection algorithm with the Akaike information criterion (AIC) was used. Variables that turned out to be very rare among the analyzed cohort were also excluded from the analysis. Model parameters were estimated using the maximum likelihood method.

In order to assess the quality of the model and control its degree of fit to the data, the considered set of observations was randomly divided into the training (70%) and testing (30%) parts. The quality of the developed classifiers was assessed using the AUC (Area Under ROC Curve) measure.

All analyses were carried out using R 3.6.1 (RStudio 1.2.5033, open-source) and Python 3.7.1 (Spyder 3.3.2, open-source). P-values less than 0.05 were considered statistically significant.

## 3. Results

### 3.1. Readmissions to the Hospital in the Period of 90 Days after Discharge

**Figure 1:**
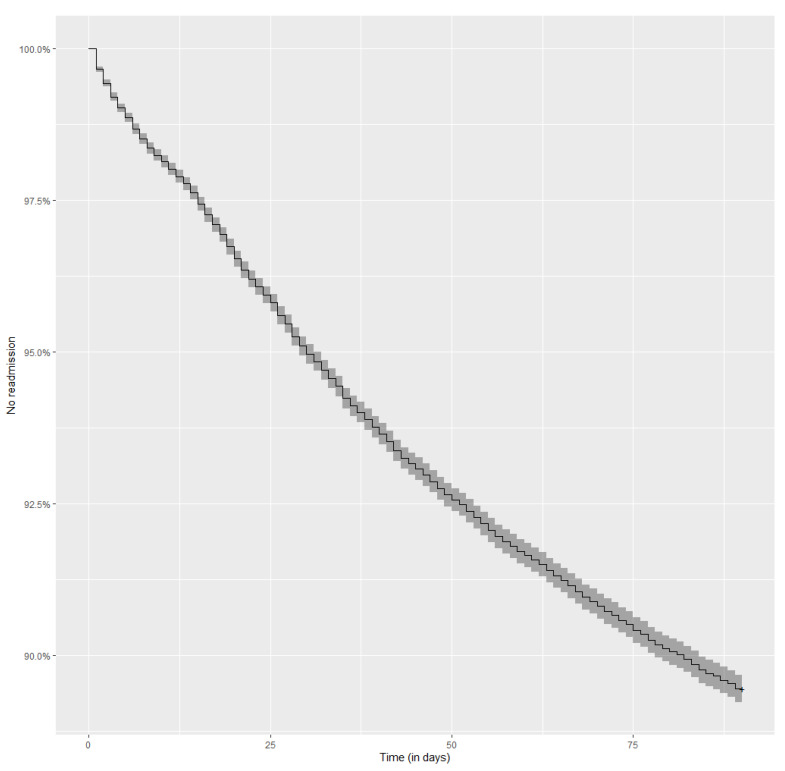
Kaplan–Meier curve representing the risk of readmission over a period of 90 days after a discharge from a diabetes-related index hospitalization. No readmission—% of patient who were NOT readmitted to the hospital over a period of 90 days after discharge from a diabetes-related index hospitalization.

In 2017, a total of 74,248 hospitalizations related to diabetes were reported to the National Health Fund. Analysis of readmissions included 69,239 hospitalizations. In this cohort, 7832 hospitalizations (11.3%) ended with readmission within 90 days of discharge. The Kaplan–Meier curve illustrating readmissions during the analyzed period is presented in Figure 1.

A logistic regression model was performed to assess the risk of readmission within 90 days of discharge. The model was properly built and the results can be generalized to the diabetic patient population (*p*-value for Hosmer–Lemeshow test > 0.05; AUC for the testing set 0.668).

The average age of patients who were rehospitalized was approximately 61.93 years (±16.77). Initial analysis of age showed a nonlinear relationship between age and the rate of rehospitalization. Given this complex relationship, it was decided to divide the age into three categories: up to 35 years, 36 to 50 years, and over 50 years. Compared with age up to 35 years, age over 50 years was associated with a lower rehospitalization risk (OR 0.87; *p* < 0.01). Men were statistically significantly more often rehospitalized than women (*p* < 0.01).

The variables involved in the annual medical history of a patient that significantly increased the chance of rehospitalization up to 90 days after hospitalization related to diabetes included hospitalization due to acute diabetic complications prior to index hospitalization (*p* < 0.01), weight loss (*p* < 0.01), peripheral artery disease (*p* < 0.01), iron deficiency anemia (*p* < 0.01), kidney failure (*p* < 0.01), alcohol abuse (*p* < 0.01), and heart failure (*p* < 0.01).

The variables involved in the index hospitalization that increased the chance of rehospitalization during the period of 90 days were determined to be urgent and emergency admission (*p* = 0.026), weekend admission (*p* = 0.010), one-day hospitalization (*p* < 0.01), hospitalization for 8–14 days (*p* < 0.01), hospitalization for longer than 14 days (*p* < 0.01) (results relating to the length of hospitalizations were compared with hospitalizations which lasted 2–7 days), and hospitalization in a teaching hospital with an endocrinology or diabetology service (*p* < 0.01). However, hospitalization in a hospital with an endocrinology or diabetology service was associated with a lower rehospitalization risk (*p* < 0.01). 

**Figure 2:**
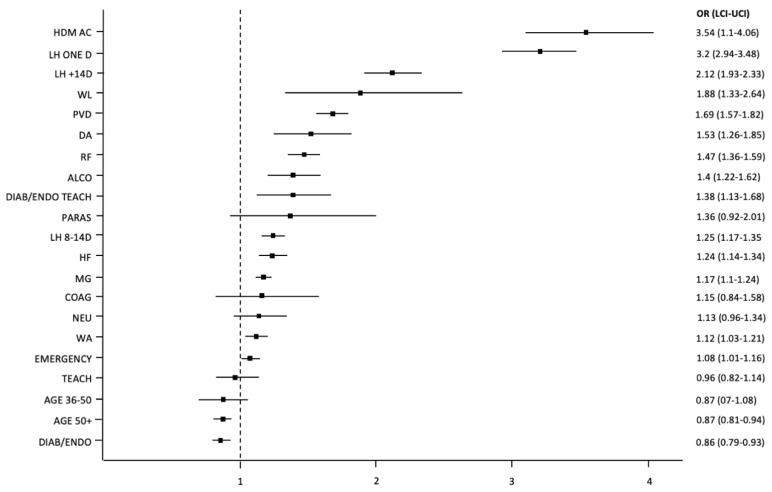
Odds ratios with 95% confidence intervals (represented by bars) for the variables used in the model that may impact readmissions to the hospital within 90 days after discharge. Abbreviations: ALCO—diseases related to alcohol abuse; AGE 36–50—patient of age 36–50; AGE 50+—patient over the age 50; COAG—coagulation disorders, purpura, and others hemorrhagic diatheses; DA—iron deficiency anemia; DIAB/ENDO—a department of diabetes or endocrinology in a center or a hospital that has a diabetology or an endocrinology contract; DIAB/ENDO TEACH—a teaching hospital with diabetology/endocrinology ward and contract; EMERGENCY—emergent or urgent mode of admission; HDM AC—hospitalization for acute complications of diabetes within 365 days prior to the index admission; HF—heart failure; LCI—lower confidence interval; LH ONE D—length of hospitalization: one day; LH 8–14 D—length of hospitalization: 8–14 days; LH +14 D—length of hospitalization: longer than 14 days; MG—male gender; NEU—other neurological diseases; OR—odds ratio; PARAS—paralytic syndromes; PVD—peripheral vascular diseases; RF—renal failure; TEACH—teaching hospital; UCI—upper confidence interval; WA—admission on the weekend; WL—malnutrition or unintended weight loss.

Odds ratios with 95% confidence intervals for the variables that may impact the rate of readmissions to the hospital in the period of 90 days after discharge are presented below (see Figure 2).

### 3.2. Death during 90 Days following Discharge

**Figure 3:**
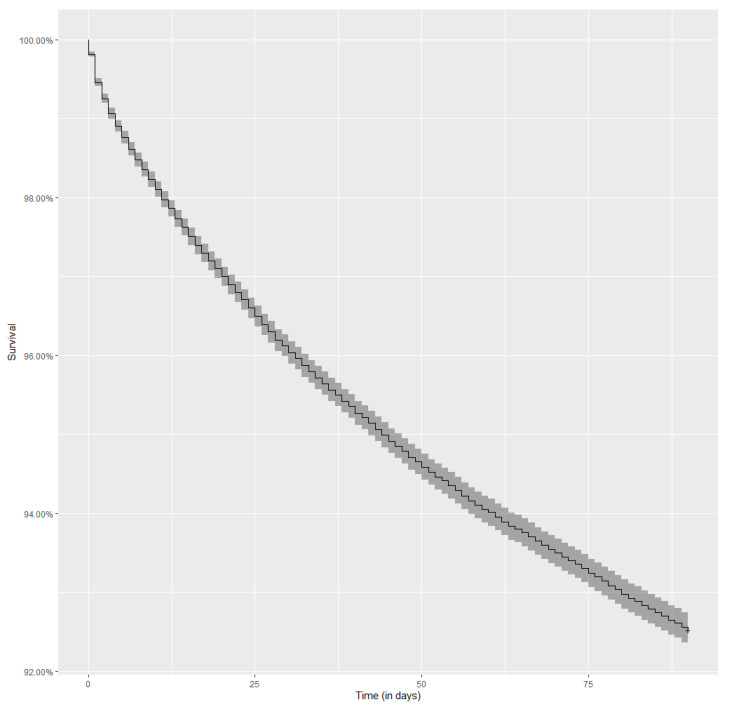
Kaplan–Meier curve representing the risk of death over a period of 90 days after a discharge from a diabetes-related index hospitalization.

A total of 74,111 hospitalizations related to diabetes were included in this analysis. Interestingly, 5412 hospitalizations (7.3%) resulted in death within a period of 90 days following discharge from the index hospitalization. The Kaplan–Meier curve illustrating patient survival during the analyzed period is displayed in Figure 3.

A logistic regression model was performed to assess the risk of death within 90 days of discharge. The model was properly built and the results can be generalized to the diabetic patient population (*p*-value for Hosmer–Lemeshow test > 0.05; AUC for the testing set 0.818).

Age significantly increased the chance of death within 90 days of discharge. The average age of patients who died during this period was 75.04 years (±12.18). An increase in age by one standard deviation (i.e., approximately 16.8 years) increased the chance of dying by 2.47 times (*p* < 0.01).

The variables involving the annual medical history of the patient that significantly increased the chance of death within 90 days following discharge included neoplastic disease with metastases (*p* < 0.01); weight loss (*p* < 0.01); neoplastic disease without metastases (*p* < 0.01); coagulation disorders (*p* < 0.01); alcohol abuse (*p* < 0.01); hospitalization due to acute diabetes complications (*p* < 0.01); heart failure (*p* < 0.01); kidney failure (*p* < 0.01); iron deficiency anemia (*p* < 0.01); peripheral artery disease (*p* < 0.01); and disorders of fluid, electrolyte, and acid–base balance (*p* < 0.01).

The variables concerning the index hospitalization that increased the chance of death during a period of 90 days following discharge included emergency admission (*p* < 0.01), urgent admission (*p* < 0.01), weekend admission (*p* = 0.01), hospitalization for 8–14 days (*p* < 0.01), and hospitalization for longer than 14 days (*p* < 0.01). One-day hospitalization and the level of experience of the admitting center were associated with a lower risk of death within 90 days following discharge (*p* < 0.01). Results relating to length of hospitalizations were also compared with hospitalizations that lasted 2–7 days. 

**Figure 4:**
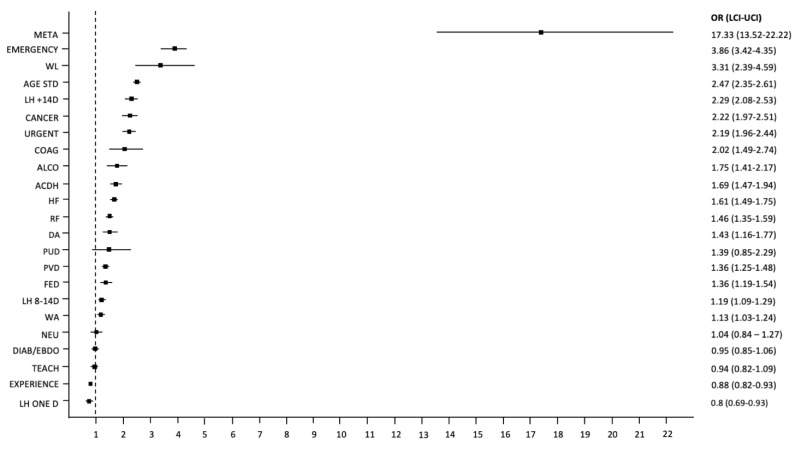
Odds ratios with 95% confidence intervals (represented by bars) for the variables used in the model that may impact the risk of death during the 90 days following discharge. Abbreviations: ACDH—hospitalization due to acute complications of diabetes; AGE STD—standardized age with the mean and standard deviation; ALCO—diseases related to alcohol abuse; CACER—solid tumors without metastases; COAG—coagulation disorders, purpura, and others hemorrhagic diatheses; DA—iron deficiency anemia; DIAB/ENDO—a department of diabetes or endocrinology in a center or hospital that has a diabetology or endocrinology contract; EXPERIENCE—experience of the center; FED—fluid, electrolyte, and acid–base imbalance; HF—heart failure; LH ONE D—length of hospitalization: one day. LH 8–14 d—length of hospitalization: 8–14 days; LH +14 d—length of hospitalization: longer than 14 days; LCI—lower confidence interval; META—metastatic tumors or carcinoma of unknown primary cause; Emergency—emergent or urgent mode of admission; NEU—other neurological diseases; OR—odds ratio; PUD—peptic ulcers without hemorrhage and perforation; PVD—peripheral vascular diseases; RF—renal failure; TEACH—teaching hospital; UCI—upper confidence interval; URGENT—mode of admission; WA—admission on the weekend; WL—malnutrition or unintended weight loss.

Odds ratios with 95% confidence intervals for the variables used in the model, which may impact the risk of death during a period of 90 days following discharge, are presented below (see Figure 4).

## 4. Discussion

In this paper, we analyzed predictors of two predefined outcomes of hospitalization based on a database comprising a large group of diabetic hospitalizations. The outcomes consisted of readmission within 90 days of discharge, and death within 90 days of discharge.

The hospital readmission rate, although commonly used as an outcome measure and quality benchmark in healthcare, indicates the financial burden that rehospitalization causes. This burden has contributed to making the reduction of the frequency of rehospitalization a critical objective for hospital leaders. We found that the predictors of readmission to the hospital within 90 days of discharge in diabetic patients are as follows: age below 35 years, male gender, acute diabetic complications prior to the index hospitalization, weight loss, some comorbidities, urgent and emergency admission, weekend admission, length of stay, hospitalization in a teaching hospital with an endocrinology or diabetology service, and hospitalization in a hospital without an endocrinology or diabetology service. The number of protective factors was much lower. Among all the studied variables, only age over 50, female gender, and a stay in the hospital with an endocrinology and diabetology service decreased the risk of rehospitalization.

It is a little surprising that age over 50 (compared to age under 35) was associated with a lower rate of rehospitalization. This may be because there are more patients with type 1 diabetes in the younger age group and patients with type 1 diabetes more frequently experience severe hypoglycemia or hyperglycemia, possibly due to the inherent nature of this disease. Additionally, young people are more often unconcerned about their illness or strict adherence to their treatment plan. Current literature shows conflicting results regarding age as a predictor of rehospitalization in internal medicine patients and in the general population. Similar to our findings, Kaya et al. [11] showed that the risk of readmission to an internal medicine ward within 3 days of discharge is inversely related to the patient’s age. Retrospective analysis of a large US database, by Berry et al. [12], found that when considering all conditions, the readmission rate increased in young adults peaked by middle age and decreased at age 65 years. In turn, in a recently published systemic review and meta-analysis, age ≥65 predicted unplanned hospital readmissions within 30 days of discharge among adult patients with diabetes mellitus [13].

In literature, gender as a risk factor for rehospitalization is disputed. In our paper, male gender was a predictor of readmission within 90 days of discharge. Similarly, Soh et al. showed that male gender is the predictor of 30-day unplanned hospital readmission among adult patients with diabetes mellitus [13]. However, in the papers of Chen et al. [14] and Karunakaran et al. [15], gender was not a significant predictor of 30-day hospital readmission in diabetes patients discharged from hospitals.

The greater burden of comorbidities is one of the major risk factors for readmission in diabetic patients [16]. In the paper of Karunakaran et al. [15], comorbidities including schizophrenia or mood disorders, gastroparesis, cardiac dysrhythmias, anemia, and fluid or electrolyte disorders were independently associated with a significantly increased risk of rehospitalization within 30 days, while Soh et al. [13] concluded that heart failure and renal disease were stronger predictors. We found that readmission within 90 days of discharge depended most strongly on acute diabetic complications prior to index hospitalization, followed by peripheral artery disease, iron deficiency anemia, kidney failure, alcohol abuse, and heart failure. The risk of such rehospitalization was also dependent on weight loss, which is consistent with the findings of Mudge et al. [17]. 

The meta-analysis performed by Soh et al. [13] showed that a longer index admission is associated with an increased risk of 30-day readmission in diabetic patients. Accordantly, we found that compared with 1–7 days, hospitalizations of 8–14 days and longer than 14 days increased the chance of rehospitalization during the period of 90 days. However, in our study, a very short (one-day) hospitalization was also a predictor of readmission. Patients with a longer length of stay tend to be in a more severe condition, have more comorbidities, and have a higher risk of nosocomial infections, all contributing to a higher rehospitalization rate. In turn, a shorter hospitalization may limit the opportunity for patient education and treatment optimization. Diabetes education prior to discharge is associated with a lower risk of readmission [18]. 

Variables regarding a patient’s hospitalization that increased the chance of readmission during the period of 90 days were also urgent, emergency, or weekend admissions. Most weekend hospitalizations are also urgent or emergency, therefore, these variables may be related to each other; however, these were independent risk factors for rehospitalization. Urgent and emergency admissions as rehospitalization risk factors have been reported in other studies as well, including in nondiabetic patients [19,20,21]. It is possible that in such patients, doctors may be focusing on a more life-threatening problem than diabetes. Lack of patient counselling and follow-up planning may increase the risk of readmission. Urgent and emergency admission patients are also often referred for hospital follow-ups for the diagnostics and treatment of other conditions.

Our study showed that readmission may be related to hospital-specific factors. Diabetic hospitalization in a teaching hospital with an endocrinology or diabetology service increased the risk of rehospitalization within 90 days, whereas a nonteaching hospital with the same services was associated with lower risk. This discrepancy may be due to the fact that teaching hospitals admit patients in a more severe condition or have more capabilities to investigate further, thereby warranting subsequent readmissions later on. Generally, it appears that the involvement of diabetes specialists may reduce the readmission rate [22,23] but not all data are consistent with this [24]. 

We found that 7.3% of hospitalizations related to diabetes resulted with death within 90 days following discharge. Diabetes increases the risk of various adverse events [25,26] and, in most papers, hospitalized patients with diabetes had greater mortality than those without diabetes [27,28]. However, the data available on postdischarge outcomes in this population of patients are scarce and, consequently, it is difficult to compare our results with those of others.

Consistent with the results of the systemic review by Mukherjee et al. [29], age significantly increases the chance of death after hospitalization, likely due to multiple comorbidities and a worse condition associated with ageing. 

We also found that variables attributed to the annual medical history of a patient that significantly increase the chance of death within 90 days following discharge were as follows: hospitalization from acute diabetes complications; weight loss; and disorders of fluid, electrolytes, and acid–base balance. Many of these are associated with an increased mortality in nondiabetic patients as well [30]. All considered, it appears that in high-risk patients, such as those hospitalized because of acute diabetes complications, those with disorders of fluid, electrolyte, and acid–base balance, as well as those with weight loss, good coordination of postdischarge follow-up care, and management might be very important. 

In our study, the risk of death after discharge in diabetic patients was also higher in those with neoplastic disease with and without metastases, coagulopathies, alcohol abuse, heart failure, kidney failure, iron deficiency anemia, and peripheral artery disease. Similarly, in a prognostic model by Levine et al., risk factors independently associated with 1-year mortality of older adults after discharge included metastatic cancer, congestive heart failure, peripheral vascular disease, renal disease, hematologic or solid, and non-metastatic malignancy [31]. Diabetic patients may find it overwhelming to adhere to treatment of their other comorbidities along with their diabetes-specific treatment and, thus, may neglect those other conditions, causing ineffective control of their illnesses and increasing their risk of mortality [32]. 

Special attention should be paid to patients with renal failure. Prevention of morbidity and mortality (especially cardiovascular) is at the center of modern therapy for diabetic kidney disease. In a recently published randomized multicenter clinical trial involving type 2 diabetes patients with diabetic kidney disease, it was shown that an intensive, multifactorial treatment of the main cardiovascular risk factors significantly reduced the risk of major cardiovascular events and all-cause mortality [33]. This strategy, in addition to the use of multitarget drugs such as sodium–glucose cotransporter 2 (SGLT2) inhibitors and glucagon-like peptide-1 (GLP-1) receptor agonists, represents the future of the therapy of this group of patients.

Additionally, we identified hospital-specific variables that increase the chance of death within 90 days of discharge, which included emergency and urgent admission as well as weekend admission. Such findings are consistent with those of other studies performed in the general population [10,34,35]. This relationship may be attributed to the fact that patients admitted as an emergency or on the weekend are in a more severe condition.

Another hospital-specific predictor of mortality after discharge was hospitalization longer than 8 days. The relationship between the length of hospital stay and increased mortality after discharge was also studied in nondiabetic patients [36,37,38]. Prolonged hospitalization typically involves patients admitted in a poorer medical state and is also associated with a propensity for in-hospital complications such as infections, thereby possibly contributing to a rise in postdischarge mortality.

We found only two protective factors against death within 90 days following discharge. The risk of death was lower in the case of one-day hospitalization and was also lower if the admitting center had a higher level of experience.

Our study has several limitations, which include the lack of information regarding the patient’s condition at discharge, laboratory values or test results, description of treatment, use of insulin, hemoglobin A1C, hypoglycemia events, blood glucose at admission, and diabetes complications. These limitations are related to the retrospective nature of the study and to the lack of the abovementioned data in the analyzed database. However, our database included other variables that have been analyzed as predictors of rehospitalization and death after discharge, for the first time in the diabetes population. Additionally, the errors inherent to a retrospective study are balanced out by the large population.

In summary, this is the first analysis of a large national database to assess the prognostic factors independently associated with rehospitalization and death during a period of 90 days postdischarge in all diabetes-related hospital admissions. The strength of this study is the fact that these data were collected from a large national database, which records information about almost all hospitalizations in Poland.

## 5. Conclusions

There are several comorbidity, demographic, and hospital-related factors that are independently associated with an increased risk of rehospitalization and death within 90 days postdischarge. However, the admission of diabetes patients to an experienced specialist center may reduce the frequency of these adverse outcomes.

## Figures and Tables

**Table 1:** Description of the variables used.

Variable.	Description	Reference Group	Definition
Medical History			
ALCO	Diseases related to alcohol abuse	No ALCO	F10, E52, G62.1, I42.6, K29.2, K70.0, K70.3, K70.9, T51, Z50.2, Z71.4, Z72.1
ACDH	Hospitalization due to acute complications of diabetes	Hospitalization not caused by acute complications of diabetes	E10.0, E11.0, E12.1, E13.0, E14.0, E10.1, E11.1, E12.1, E13.1, E14.1
BLA	Anemia due to chronic blood loss	No BLA	D50.0
CACD	Cardiac arrhythmias and conduction disturbances	No CACD	I44.1, I44.2, I44.3, I45.6, I45.9, I47, I48, I49, R00.0, R00.1, R00.8, T82.1, Z45.0, Z95.0
Cancer	Solid tumors without metastases	No cancer and/or metastases	C00, C01, C02, C03, C04, C05, C06, C07, C08, C09, C10, C11, C12, C13, C14, C15, C16, C17, C18, C19, C20, C21, C22, C23, C24, C25, C26, C30, C31, C32, C33, C34, C37, C38, C39, C40, C41, C43, C45, C46, C47, C48, C49, C50, C51. C52, C53, C54, C55, C56, C57, C58, C60, C61, C62, C63, C64, C65, C66, C67, C68, C69, C70, C71, C72, C73, C74, C75, C76, C97
COAG	Coagulation disorders, purpura, and others hemorrhagic diatheses	No COAG	D65, D66, D67, D68, D69.1, D69.3, D69.4, D69.5, D69.6
CD-LRT	Chronic diseases of the lower respiratory tract	No CD-LRT	I27.8, I27.9, J40, J41, J42, J43, J44, J45, J46, J47, J60, J61, J62, J63, J64, J65, J66, J67, J68.4, J70.1, J70.3
DA	Iron deficiency anemia	No DA	D50.8, D50.9, D51, D52, D53
DM-INS	Diabetes and hyperglycemic conditions treated with Insulin	Lack of diabetes and hyperglycemic conditions	Purchase of insulin (at least once) by the patient within a year before hospitalization
DM	Diabetes and hyperglycemic conditions treated with medication or controlled by diet	Lack of diabetes and hyperglycemic conditions	Purchase (at least once) by the patient of antidiabetic drugs or test strips in the year prior to hospitalization and, at the same time, not buying insulin within a year before hospitalization
DEP	Depression and depressive disorders	No DEP	F20.4, F31.3, F31.4, F31.5, F32, F33, F34.1, F41.2, F43.2
DRUG	Drug use	No DRUG	F11, F12, F13, F14, F15, F16, F18, F19, Z71.5, Z72.2
FED	Fluid, electrolyte and acid-base imbalance	No FED	E22.2, E86, E87
HDM-AC	Hospitalization for acute complications of diabetes within 365 days prior to index admission	No hospitalization due acute complications of diabetes in the annual medical history of the patient	
HF	Heart failure	No HF	I09.9, I11.0, I13.0, I13.2, I25.5, I42.0, I42.5, I42.6, I42.7, I42.8, I42.9, I43, I50, P29.0
HIV	Disease caused by Human Immunodeficiency Virus (HIV)	No HIV	B20, B21, B22, B24
HTH	Subclinical and clinical hypothyroidism	No HTH	E00, E01, E02, E03, E89.0
HTC	Complicated or secondary hypertension	No HTC or HTU	I11, I12, I13, I15
HP	Primary hypertension	No HTC or HTP	I10
LD	Liver diseases and their complications	No LD	B18, I85, I86.4, I98.2, K70, K71.1, K71.3, K71.4, K71.5, K71.7, K72, K73, K74, K76.0, K76.2, K76.3, K76.4,
LYMP	Malignant tumors of the lymphatic, hematopoietic, and related systems	No LYMP	C81, C82, C83, C84, C85, C88, C96, C90.0, C90.2
META	Metastatic tumors or carcinoma of unknown primary	No cancer and/or metastases	C77, C78, C79, C80
NEU	Other neurological diseases	No NEU	G10, G11, G12, G13, G20, G21, G22, G25.4, G25.5, G31.2, G31.8, G31.9, G32, G35, G36, G37, G40, G41, G93.1, G93.4, R47.0, R56
OBE	Obesity	No OBE	E66
PARAS	Paralytic syndromes	No PTS	G04.1, G11.4, G80.1, G80.2, G81, G82, G83.0, G83.1, G83.2, G83.3, G83.4, G83.9
PCD	Pulmonary circulation disorders	No PCD	I26, I27, I28.0, I28.8, I28.9
PSYCH	Psychotic disorders	No PSYCH	F20, F22, F23, F24, F25, F28, F29, F30.2, F31.2, F31.5
PUD	Peptic ulcers without hemorrhage and perforation	No PUD	K25.7, K25.9, K26.7, K26.9, K27.7, K27.9, K28.7, K28.9
PVD	Peripheral vascular diseases	No PVD	I70, I71, I73.1, I73.8, I73.9, I77.1, I79.0, I79.2, K55.1, K55.8, K55.9, K95.8, Z95.9
RF	Renal failure	No RF	I12.0, I13.1, N18, N19, N25.0, Z49.0, Z49.1, Z49.2, Z94.0, Z99.2
RHEU	Arthropathies and connective tissue diseases	No RHEU	L94.0, L94.1, L94.3, M05, M06, M08, M12.0, M12.3, M30, M31.0, M31.1, M31.2, M31.3, M32, M33, M34, M35, M45, M46.1, M46.8, M46.9
VD	Heart valve defects	No VD	A52.0, I05, I06, I07, I08, I09.1, I09.8, I34, I35, I36, I37, I38, I39, Q23.0, Q23.1, Q23.2, Q23.3, Z95.2, Z95.3, Z95.4
WL	Malnutrition or unintended weight loss	No WL	E40, E41, E42, E43, E44, E45, E46, R63.4, R64
Demographics			
Place of residence	Patient’s place of residence—rural/city	City	
MG	Male gender	Female	
Hospitalization			
Experience	Experience of the center		Percentage of diabetes hospitalizations carried out by the center relative to all hospitalizations due to diabetes in Poland standardized by a mean and standard deviation
Diab/Endo	A department of diabetes or endocrinology in a center or hospital that has a diabetology or endocrinology service	A facility that does not have a diabetology or endocrinology ward	
Diab/Endo Teach	Teaching hospital with diabetology/endocrinology ward and service	A nonteaching facility or no services in the field of diabetology or endocrinology	
Teach	Category of hospitalization facility: teaching	A nonteaching facility	
LH one_d	Length of hospitalization: one day	Patient was discharged between the 1st and 7th day from the date of admission	Date of the patient’s discharge is the same as the date of admission
LH 1–7 d	Length of hospitalization: 1–7 d		Patient was discharged between the 1st and 7th day from the date of admission
LH 8–14 d	Length of hospitalization: 8–14 d	Patient was discharged between the 1st and 7th day from the date of admission	Patient was discharged between the 8th and 14th day from the date of admission
LH +14 d	Length of hospitalization: +14 d	Patient was discharged between the 1st and 7th day from the date of admission	Patient was discharged at least 15 days from the date of admission
Secondary hospitalization	Secondary hospitalization	Hospitalization is not secondary	
WA	Admission on weekend	Admission between Monday and Thursday	Admission on Friday, Saturday, or Sunday

**Table 2:** Variables that were used only for modeling rehospitalization within 90 days after discharge.

Variable	Description	Reference Group
Demographics		
Age 36–50	Patient of age 36–50	Adult patient below the age 36
Age 50+	Patient over the age 50	Adult patient below the age 36
Hospitalization		
Emergency	Emergency or urgent mode of admission (emergency—patient was brought by ambulance, urgent—other urgent admissions)	Planned admission

**Table 3:** Variables that were used only for modeling the risk of death within 90 days of discharge.

Variable	Description	Reference Group
Demographics		
Age std	Standardized age with the mean (avg = 63.26) and standard deviation (std = 16.78)	
Age std sq	Standardized age with the mean (avg = 63.26) and standard deviation (std = 16.78) squared	
Hospitalization		
Emergency	Mode of admission (patient brought by ambulance)	Planned admission
Urgent	Mode of admission	Planned admission

## Data Availability

The data presented in this study are available on request from the corresponding author. The data are not publicly available due to privacy.
